# Psychometric validation of the brief Chinese psychological well-being scale in a sample of Mainland Chinese adolescents

**DOI:** 10.1186/s40359-025-03952-x

**Published:** 2026-01-09

**Authors:** Yicen Meng, Nik Rosila Nik Yaacob, Yasmin Othman Mydin, Fengjie Li

**Affiliations:** 1 School of Educational Studies, Universiti Sains Malaysia, George Town, 11800 Penang Malaysia; 2 Dalian Academy of Education, Dalian, 116021 China

**Keywords:** Psychological well-being, Brief PWBS, Internal consistency, Confirmatory factor analysis, Measurement invariance, Chinese adolescents

## Abstract

**Background:**

Ryff’s Psychological Well-Being Scale (PWBS) has been widely used to assess eudaimonic well-being in adults and older populations within the framework of positive psychology. However, concerns have been raised regarding its length, factorial structure, and suitability for younger groups, particularly Chinese adolescents. While several abbreviated versions in different languages exist, few were specifically designed for adolescent samples, and many have demonstrated inadequate model fit and internal reliability. The brief Chinese PWBS version (BC-PWBS), developed by Chan et al. (2019), was translated and adapted for use in Chinese populations. While prior studies have supported its reliability and validity in Hong Kong adults and adolescents, its psychometric performance among adolescents in Mainland China has not yet been thoroughly investigated.

**Methods:**

A total of 855 Chinese adolescents aged 15–18 years in a sample of Northeast China completed the 24-item BC-PWBS. Internal consistency was evaluated through Cronbach’s α and McDonald’s ω. Construct validity was assessed via confirmatory factor analysis and convergent/discriminant indices (CR and AVE). Criterion-related validity was determined by correlating BC-PWBS scores with the Academic Self-Efficacy Scale (ASES). Multi-group CFA was employed to test measurement invariance across gender and age categories. Participants completed the 24-item scale in a single administration.

**Results:**

The BC-PWBS demonstrated excellent reliability (Cronbach’s α and McDonald’s ω > 0.95). CFA supported the theorized six-factor model with acceptable fit (CFI = 0.93, TLI = 0.92, RMSEA = 0.069 [90% CI = 0.066–0.073], SRMR = 0.046). Criterion validity was supported by significant positive correlations between the BC-PWBS and the Academic Self-Efficacy Scale (ASES), consistent with theoretical expectations. Furthermore, the scale exhibited configural, metric, and scalar invariance across gender and age groups, indicating structural stability and supporting its appropriateness for subgroup comparisons.

**Conclusions:**

The present findings offer strong evidence for the psychometric soundness of the BC-PWBS among mainland Chinese adolescents, supporting its use as a concise measure for assessing psychological well-being in this population and establishing a foundation for future cross-cultural and developmental research.

**Supplementary Information:**

The online version contains supplementary material available at 10.1186/s40359-025-03952-x.

## Introduction

The expanding influence of positive psychology has led to heightened scholarly focus on the construct of well-being [1–3]. A strong sense of well-being benefits not only individuals but also broader society [4, 5]. Scholars investigating human well-being generally distinguish between two primary conceptual frameworks: the hedonic and eudaimonic perspectives. The hedonic approach centres on the pursuit of pleasure and positive emotional experiences, often reflected in measures of *subjective well-being* through indicators like happiness and life satisfaction. In contrast, the eudaimonic perspective emphasizes self-realization and the achievement of one’s full potential, focusing on aspects like meaning, purpose, and optimal psychological functioning—collectively described as *eudaimonic well-being* [2].

A well-established framework for operationalizing eudaimonic well-being is Ryff’s six-factor model of *psychological well-being* (PWB) [6]. This model asserts that genuine well-being stems not solely from positive feelings but from realizing one’s potential across six core dimensions: *autonomy*,* environmental mastery*, *personal growth*, *positive relations with others*, *purpose in life*, and *self-acceptance*. Ryff’s multidimensional approach has been broadly validated in diverse populations and has become a foundational model in global well-being research [7, 8]. It has also demonstrated cross-cultural applicability (e.g., [9, 10]) and underpins the PWB Scale (PWBS), which have been developed in multiple formats, ranging from 20 items per domain to brief versions with as few as 3 items per dimension [6, 11].

While the original 120-item form provides comprehensive coverage, its considerable length presents practical challenges, particularly in large-scale or time-constrained settings. In response, researchers have developed various shortened versions with 14 [11], 7 [12], 4 [13], and even 3 items per dimension [14, 15]. While abbreviated forms of the scale reduce respondent burden and enhance completion rates, several studies have noted a decline in internal consistency, particularly within individual subscales (e.g., [1, 16]). One noteworthy deviation to this trend is Li’s 15-item Chinese adaptation, which showed satisfactory reliability and model fit in a large adult sample from Taiwan [15].

Beyond psychometric concerns, another challenge relates to age-appropriateness. Most validations of Ryff’s scales have been conducted with adults or older populations [17], raising questions about their suitability for adolescents. Evidence suggests that responses to PWBS items may vary significantly by age and gender [18], and factor structures may not be stable across groups (e.g., [13]). Existing Chinese short forms have mainly been validated among older adult samples, with mean ages of 59.8 [15] and 44.7 [13]. Consequently, the psychometric properties of the scale among adolescents remain largely unexamined.

In response to these limitations, Chan et al. developed a 24-item brief adaptation of Ryff’s PWBS specifically designed for adolescents in Hong Kong [19]. Building on the original six-dimension model, they employed a two-stage factor analytic strategy: an initial exploratory factor analysis (EFA) identified a concise item pool, confirmatory factor analysis (CFA) was then performed on a separate sample to verify the factorial structure. Their final brief scale demonstrated robust internal consistency across all six subscales (Cronbach’s α ranging from 0.77 to 0.88) and acceptable fit indices in the CFA (CFI ≥ 0.93; RMSEA ≤ 0.06), supporting retention of the theoretical six-factor model in a Hong Kong adolescent cohort. Their version emphasized linguistic simplicity and age-appropriate clarity. Since its initial validation, the 24-item brief Chinese PWBS version (BC-PWBS) by Chan et al. has been employed in various applied studies, further supporting its reliability. For example, Sun et al. administered the same adolescent version to 277 secondary students in Hong Kong, reporting subscale reliabilities ranging from α = 0.72 to 0.85 and confirming the six-factor model through CFA [20]. More recently, Lee et al. used the BC-PWBS in a randomized controlled trial evaluating a positive psychology intervention for parents of young children [21]. In this adult sample, the six four-item subscales demonstrated strong internal consistency, with Cronbach’s α values ranging from 0.81 to 0.95 across both baseline and post-intervention assessments. Despite these promising results, the scale’s generalizability beyond the Hong Kong context remains uncertain. To date, no validation studies have examined the adolescent brief version in Mainland China, where cultural and linguistic variations may affect item interpretation and factor structure. Furthermore, measurement invariance across key demographic groups has yet to be thoroughly investigated.

Despite the lack of evidence from mainland China, prior research in other countries has explored the measurement invariance (MI) of the PWBS and its short forms across various demographic or cultural groups. For instance, Stavraki et al. validated a short version of the PWBS among Spanish adolescents and confirmed MI across gender and age groups [22], while Sunardy et al. demonstrated age-based invariance of the Indonesian Brief Scale of Psychological Well-Being for Adolescents [23]. In addition, Sirigatti et al. established cross-national measurement invariance of the original PWBS between Italian and Belarusian samples, showing that the construct of psychological well-being is comparably understood across different cultural contexts [24]. Similarly, Garcia et al. examined the psychometric properties of the Swedish 18-item version of the PWBS using both classical test theory and item response theory and confirmed gender-based measurement invariance [14]. Within the Chinese context, Xia and Li developed and validated a psychological well-being scale specifically for undergraduates, providing valuable insights into well-being measurement in higher education populations [25]. However, no published study has yet tested the measurement invariance of the Brief Chinese PWBS (BC-PWBS) among adolescents in mainland China. Establishing such evidence is crucial for ensuring that the construct of psychological well-being is interpreted equivalently across demographic groups and developmental stages, and for supporting its use in youth well-being research and practice [26, 27].

To bridge these gaps, the present study sought to validate the 24-item Brief Chinese Psychological Well-Being Scale (BC-PWBS) adapted for adolescents by Chan et al. among high school students aged 15 to 18 in a sample of Mainland China [28]. Throughout the paper, we refer to this instrument as the BC-PWBS for brevity. Specifically, we assessed its internal consistency, factorial structure, criterion-related validity, and measurement invariance across gender and age groups. Academic self-efficacy is widely regarded as an important psychological resource that contributes to adolescents’ well-being. According to Bandura’s self-efficacy theory, individuals with stronger beliefs in their academic capabilities experience greater motivation, competence, and mastery, which are integral components of psychological well-being [29]. Within the framework of self-determination theory [30, 31], academic self-efficacy also fulfils the three basic psychological needs that underlie well-being—competence, autonomy, and relatedness. Students who feel efficacious in their academic pursuits experience a stronger sense of competence in mastering school tasks, greater autonomy in regulating their learning behaviour, and enhanced relatedness through positive interactions with teachers and peers. These experiences jointly contribute to adolescents’ sustained motivation and psychological well-being. Given that academic success and self-perceived competence are central to adolescents’ daily experiences, the Academic Self-Efficacy Scale (ASES) was selected as the criterion variable to examine the concurrent association between academic confidence and psychological well-being in this study. This validation effort was intended to establish the BC-PWBS as a reliable and practical instrument for assessing psychological well-being in educational research settings. Given the increasing emphasis on adolescent mental health and well-being in China [32, 33], establishing a psychometrically sound and developmentally appropriate measure is both timely and valuable. Validating the BC-PWBS among adolescents also contributes to the cross-cultural applicability of Ryff’s well-being framework and provides a foundation for future comparative and longitudinal studies.

## Materials and methods

### Participants

The data for this validation study were extracted from a larger doctoral research project on adolescent well-being in Mainland China, approved by the first author’s University Ethics Committee. The sample was restricted to senior high school students (Grades 10 and 11) to ensure developmental homogeneity, as substantial cognitive and contextual differences exist between junior and senior secondary students, which could influence the psychometric properties of the scale. A total of 855 valid responses were retained after data screening. Participants were included if they were currently enrolled full-time in Grade 10 and 11 of public high schools in Dalian City, Northeast China, and provided parental consent. Students in Grade 12 were excluded from the present study because they were busy preparing for the National College Entrance Examination (NCEE) and those with incomplete data or self-reported psychological diagnoses were excluded as well. The sample included 387 males (45.3%) and 468 females (54.7%), the average age was 16.41 years (SD = 0.66), ranging from 15 to 18. By grade level, 58.5% were in Grade 10 and 41.5% in Grade 11. Prior to analysis, 71 cases were excluded due to missing responses, response patterns, or statistical outliers identified via Mahalanobis distance method [34].

### Procedure

Cultural adaptation involves a systematic and rigorous process to ensure that a psychological instrument is both culturally appropriate and psychometrically valid for use in a new cultural context [35, 36]. In this study, we first examined the conceptual equivalence of Ryff’s original PWB model and confirmed that its multidimensional framework, which includes autonomy, purpose in life, personal growth, and other eudaimonic components, is theoretically suitable for Chinese adolescents based on a literature review and expert consultation. Prior studies conducted in Chinese contexts have provided theoretical support for the applicability of the PWB construct (e.g., [15, 19]). For cultural adaptation, two bilingual psychologists with expertise in adolescent development and psychometrics independently reviewed the BC-PWBS items and evaluated the construct dimensions to confirm linguistic clarity and their relevance to the cultural and developmental context of Chinese adolescents.

Before the full-scale administration, A pilot study involving 20 Chinese adolescents aged 15 to 18 was conducted to evaluate the clarity of language, cultural appropriateness, and overall comprehensibility of the questionnaire items. Participants indicated that the items were easy to understand and respond to, and therefore, no additional modifications were deemed necessary.

Ethical approval for this study was granted by the ethics committee of the first author’s university. As the researchers were not affiliated with any Chinese institutions, no additional ethics approval was sought from China, and access to participating schools was granted by local education authorities. Data were collected in classroom settings during regular school hours. Participants completed paper-and-pencil questionnaires following standardized procedures supervised by the research team and trained teachers. Prior to administration, the study’s purpose was clearly explained to all participants. Participation was entirely voluntary, and was obtained from both students and their legal guardians prior to participation. Anonymity and confidentiality of responses were fully ensured.

### Instruments

#### The brief Chinese psychological well-being scale (BC-PWBS)

The *Brief Chinese Psychological Well-being Scale* (BC-PWBS) was adapted by Chan et al. [19] from Ryff’s original 42-item scale [6]. It is developed to assess the eudaimonic well-being of adolescents. This 24-item scale comprises six dimensions: *Autonomy* (AU), *Environmental Mastery* (EM), *Personal Growth* (PG), *Positive Relations with Others* (PR), *Purpose in Life* (PL), and *Self-Acceptance* (SA). Items are rated on a five-point Likert scale from 1 (strongly disagree) to 5 (strongly agree), with higher scores indicating greater psychological well-being. Total and subscale scores are computed as the mean of their respective items. The Chinese version was administered in simplified Mandarin. Previous empirical studies have provided evidence for the scale’s reliability, with Cronbach’s α coefficients for its subscales falling between 0.67 and 0.83 [19], which indicates acceptable to good internal consistency. Additionally, CFA has demonstrated a solid model fit (χ²/df = 2.89, CFI = 0.93, RMSEA = 0.05). The brief Chinese version of the PWBS [19] was used without modification, except for converting Traditional Chinese to Simplified Chinese wording for consistency. The content and structure remained identical to the original brief Chinese version.

#### Academic self‑efficacy scale (ASES)

The *Academic Self-Efficacy Scale* (ASES) was administered to assess adolescents perceived competence in academic contexts. This measure was included for the purpose of examining criterion-related validity of the BC-PWBS, given that academic self-efficacy has been theoretically and empirically linked to adolescents’ psychological well-being [29, 30, 37, 38]. This scale was initially developed by Pintrich and De Groot [39] and later adapted for Chinese students by Liang [40], assesses learners’ confidence in their academic capabilities and study-related behaviors. Comprised of 22 items, the scale uses a 5-point Likert format for all responses, ranging from 1 (“Strongly disagree”) to 5 (“Extremely agree”), with higher scores reflecting stronger perceived academic self-efficacy (ASE). An overall score is derived by averaging the item responses. Previous studies involving Chinese adolescent populations have demonstrated the scale’s strong reliability and construct validity [28, 41]. In this current sample, the ASES showed robust internal consistency (Cronbach’s α = 0.893), reinforcing its suitability as a measure of criterion validity.

### Data analysis

We performed data analysis using SPSS (v26.0), Jamovi (v2.6), and AMOS (v26.0). Before proceeding with inferential statistics, descriptive analyses were performed for each item, including calculations of means, standard deviations, skewness, and kurtosis, to evaluate the distributional characteristics of the data. Skewness and kurtosis values falling within ± 2 and ± 7, respectively, were regarded as indicative of acceptable univariate normality [42].

First, the internal consistency of the overall scale and its subdimensions was evaluated using Cronbach’s α and McDonald’s ω coefficients. To further assess reliability, split-half analysis was conducted by dividing items into two subsets based on item order (i.e., odd- and even-numbered items). The Pearson correlation between the two halves was calculated, and the Spearman-Brown prophecy formula was used to calculate the split-half reliability of the full scale.

Second, CFA was implemented in AMOS with the maximum likelihood estimation method to examine the proposed factor structure of the PWBS [43]. Model fit was assessed using multiple indices: the chi-square to degrees of freedom ratio (χ²/df ≤ 3), Comparative Fit Index (CFI ≥ 0.90), Tucker-Lewis Index (TLI ≥ 0.90), Root Mean Square Error of Approximation (RMSEA ≤ 0.08), Standardized Root Mean Square Residual (SRMR ≤ 0.08), and the Akaike Information Criterion (AIC) for model comparison. These cutoffs were interpreted based on the established guidelines [42, 44].

Third, we calculated the average variance extracted (AVE) and composite reliability (CR) for each latent construct to evaluate convergent validity. AVE values of 0.50 or higher and CR values above 0.70 were considered evidence of adequate convergent validity. We examined whether the square root of each AVE exceeded the corresponding inter-construct correlations to assess discriminant validity. Discriminant validity was considered acceptable when the AVE square root exceeded the construct’s correlations with all other factors [45].

Consistent with previous validation studies, such as Chan et al., who used the General Self-Efficacy Scale as a benchmark when validating the brief Chinese PWBS [19], the present study employed the Academic Self-Efficacy Scale (ASES) as the criterion measure. The ASES is a validated tool that measures students perceived academic competence and has demonstrated solid psychometric properties in Chinese adolescent samples [28, 41]. Prior studies have consistently shown positive associations between ASE and PWB (e.g., [46]). To assess the criterion-related validity of the Brief Chinese PWBS, we computed Pearson correlation coefficients between the total and subscale scores of the PWBS and the ASES. In addition, a linear regression analysis was conducted to examine whether psychological well-being could significantly predict academic self-efficacy. These analyses were performed to determine both the concurrent and predictive validity of the scale.

Finally, to examine whether the PWBS factor structure remained consistent across demographic subgroups, a multi-group CFA was conducted in AMOS 26.0. Measurement invariance was assessed across gender (male vs. female) and age categories (15–16 vs. 17–18 years) through a stepwise evaluation of configural, metric, and scalar models, following the procedures outlined by Byrne and Milfont and Fischer [47, 48]. This approach was adopted because grade structures vary across school systems, whereas age provides a more developmentally and cross-culturally comparable indicator. Similar age-based grouping has been used in previous validation studies of well-being scales among adolescents (e.g., [22]), as well as with methodological recommendations emphasizing the importance of testing invariance across relevant demographic variables [26, 27]. The analysis began with an assessment of configural invariance to determine whether the underlying factor structure could be consistently identified across groups without applying equality constraints. A good model fit at the configural level indicates that the construct is conceptualized similarly across different subgroups. We then tested metric invariance by constraining factor loadings to examine whether item-factor relationships were consistent across groups. Scalar invariance was then tested by imposing constraints on both factor loadings and item intercepts. Establishing scalar invariance allows for valid comparisons of latent mean scores between groups. Model comparisons were primarily guided by changes in fit indices, particularly the comparative fit index (ΔCFI), with a change smaller than 0.01 considered evidence of invariance [49]. In cases where full scalar invariance was not supported, partial invariance was explored by freeing selected intercepts based on modification indices, as long as the revised model retained an acceptable fit [50].

## Results

Descriptive statistics were generated using SPSS to assess the distributional features of the Brief Chinese PWBS. As shown in Table 1, skewness values ranged from − 0.590 to 0.201, and kurtosis values ranged from − 0.881 to − 0.334. These statistics fall within the commonly accepted thresholds of ± 2 for assessing univariate normality [51, 52], suggesting that the data approximate a normal distribution. In addition, the mean and median scores for individual items were generally similar, further supporting the assumption of normality [53]. Corrected item–total correlations (CITC) were calculated to examine the consistency between each item and the total score of the remaining items within the same subscale. A CITC value above 0.30 generally indicates acceptable item–total consistency [54].

**Table 1:** Descriptive statistics for the brief Chinese PWBS

Items	Median	Mean	SD	Skewness	Kurtosis	CITC
PWB1	3	2.894	1.212	0.201	−0.862	0.488
PWB2	3	3.048	1.163	0.136	−0.881	0.590
PWB3	3	3.220	1.104	0.037	−0.837	0.647
PWB4	4	3.557	1.064	−0.220	−0.728	0.633
PWB5	4	3.679	0.969	−0.272	−0.437	0.643
PWB6	4	3.678	0.945	−0.152	−0.650	0.642
PWB7	4	3.615	1.020	−0.176	−0.792	0.712
PWB8	4	3.687	1.013	−0.291	−0.626	0.685
PWB9	4	3.827	0.961	−0.418	−0.375	0.643
PWB10	4	3.714	1.014	−0.386	−0.450	0.674
PWB11	4	3.868	0.930	−0.392	−0.406	0.698
PWB12	4	3.984	0.964	−0.590	−0.408	0.666
PWB13	4	3.792	0.976	−0.402	−0.419	0.568
PWB14	3	3.497	1.141	−0.332	−0.594	0.538
PWB15	3	3.380	1.061	−0.161	−0.473	0.505
PWB16	4	3.566	1.054	−0.406	−0.334	0.543
PWB17	4	3.571	1.090	−0.316	−0.666	0.716
PWB18	4	3.504	1.115	−0.222	−0.854	0.703
PWB19	3	3.357	1.135	−0.095	−0.827	0.658
PWB20	3	3.367	1.099	−0.159	−0.672	0.701
PWB21	4	3.677	1.028	−0.381	−0.449	0.671
PWB22	4	3.678	1.107	−0.471	−0.551	0.790
PWB23	4	3.732	1.126	−0.521	−0.540	0.702
PWB24	4	3.677	1.126	−0.485	−0.556	0.712
AU	3	3.1797	0.951	0.090	−0.621	
EM	3.75	3.6645	0.861	−0.083	−0.638	
PG	4	3.8482	0.801	−0.406	−0.270	
PR	3.5	3.5589	0.835	−0.241	−0.077	
PL	3.5	3.450	0.993	−0.160	−0.595	
SA	3.75	3.691	0.949	−0.407	−0.445	

*AU* Autonomy, *EM* Environmental Mastery, *PG* Personal Growth, *PR* Positive Relations with Others, *PL* Purpose in Life, *SA* Self-Acceptance. These are the dimensions of PWBS. *CITC* Corrected Item–Total Correlation

Given the approximate normality of the data, CFA was carried out using the maximum likelihood (ML) estimation method. ML is considered suitable when multivariate normality can be reasonably assumed, and it was used here to estimate factor loadings and standard errors [55].

### Internal consistency

We examined the reliability of the BC-PWBS using multiple complementary indices. The overall internal consistency was excellent, with Cronbach’s α coefficient for the total scale reaching 0.949. Subscale reliability estimates were also high: AU = 0.856, EM = 0.895, PG = 0.847, PR = 0.797, PL = 0.917, and SA = 0.887. Consistent results were observed using McDonald’s omega, with ω = 0.951 for the full scale and subscale values of 0.862 (AU), 0.896 (EM), 0.850 (PG), 0.799 (PR), 0.918 (PL), and 0.889 (SA), confirming the scale’s internal reliability [56].

Split-half reliability was also strong, yielding coefficients of 0.894 for odd-numbered items and 0.905 for even-numbered items. The Spearman-Brown adjusted reliability reached 0.972, indicating high consistency between test halves. Corrected item-total correlations ranged from 0.49 to 0.79, exceeding the commonly accepted threshold of 0.30, and removing any item did not raise Cronbach’s alpha above 0.949. These results suggest that all items contribute meaningfully to the overall scale and that none negatively affect its internal consistency. Collectively, these findings affirm the Brief Chinese PWBS as a psychometrically robust instrument for measuring psychological well-being among Chinese adolescents.

### Confirmatory factor analysis and construct validity

Validity evidence based on internal structure was examined using confirmatory factor analysis (CFA), following the *Standards for Educational and Psychological Testing* [57] in AMOS 26, with the Maximum Likelihood (ML) estimation method with bootstrapping, as recommended by Bollen and Stine [43]. We applied bootstrapping procedures to estimate standard errors, model parameters, and fit indices.

**Fig. 1:**
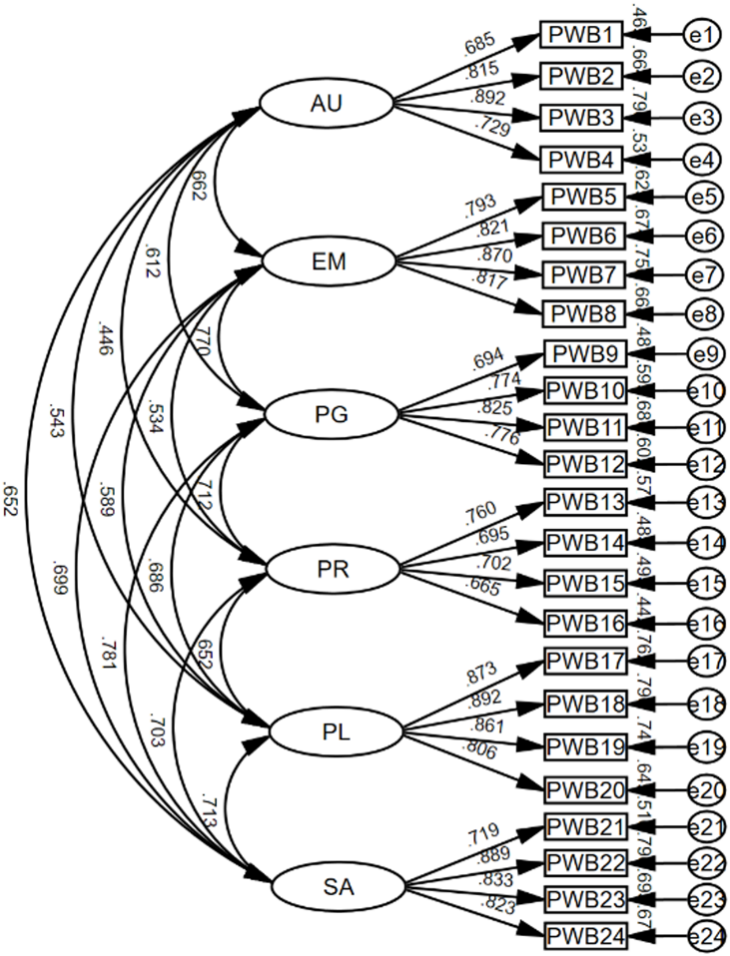
Standardized CFA path diagram of the six-factor model of brief Chinese PWBS (*N* = 855). *Note. *Abbreviations as in Table 1, PWB1–PWB24 = observed items

Following previous research [8, 13, 15], four competing models were tested: a unidimensional model, a six-factor model, a second-order (hierarchical) model, and a bi-factor model. In the unidimensional model, all 24 items were loaded onto a single latent construct. The six-factor model assigned items to their respective theoretical dimensions, allowing the six latent variables to correlate freely (see Fig. 1). The hierarchical model specified each item loading onto its corresponding factor, with all six factors loading onto a higher-order factor representing overall PWB, consistent with Ryff and Keyes’s (1995) theoretical framework. In the bi-factor model, all items loaded on a general well-being factor while also loading on their respective domain factors, capturing both shared and unique aspects of psychological well-being. A summary of model fit indices for these four structures is displayed in Table 2.

**Table 2:** The model indices of the competing CFA models

Models	χ^2^	df	CFI	SRMR	RMSEA [90%CI]	AIC
One-factor model	4458.757	252	0.701	0.085	0.140[0.136, 0.143]	4554.757
Six-factor model	1211.023	237	0.931	0.046	0.069[0.066, 0.073]	1337.023
Hierarchical model	1309.190	246	0.924	0.055	0.071[0.067, 0.075]	1417.190
Bi-factor model	847.836	214	0.955	0.113	0.059[0.055, 0.063]	1019.836

Model fit statistics revealed that the one-factor solution yielded poor fit (CFI = 0.701, RMSEA = 0.140), whereas the hierarchical model achieved a satisfactory level of fit, albeit marginally weaker than the six-factor model (CFI = 0.924, RMSEA = 0.071, AIC = 1417.19). The six-factor model demonstrated acceptable to good fit to the data: χ²(237) = 1211.02, *p* < 0.001; χ²/df = 5.11; CFI = 0.931; TLI = 0.919; RMSEA = 0.069; and SRMR = 0.046. Although the chi-square statistic was significant and the χ²/df ratio slightly surpassed the commonly recommended cutoff—likely due to the sensitivity of these indices to large sample sizes—other key fit indicators (CFI, TLI, RMSEA, and SRMR) suggested an acceptable to good model fit [42, 44]. Importantly, the bi-factor model showed the best overall fit among all tested models (χ²(214) = 847.84, CFI = 0.955, RMSEA = 0.059 [90% CI = 0.055–0.063], SRMR = 0.113, AIC = 1019.84), indicating that a general factor of psychological well-being coexisted with specific dimensions, capturing both shared and unique variance in the construct. Although the SRMR value (0.113) slightly exceeded the conventional cutoff of 0.10, suggesting some residual misfit at the item level [44]. Given the model’s complexity and satisfactory performance on other indices, the fit was deemed acceptable. As presented in Table 3, all standardized factor loadings ranged from 0.685 to 0.899, exceeding the 0.60 benchmark [58], thereby supporting satisfactory item reliability.

**Table 3:** Standardized factor loadings, CR, and AVE for the PWBS dimensions

Dimensions	Standardized factor loadings	CR	AVE	AVE’s square root
AU	0.685, 0.815, 0.892, 0.729	0.864	0.615	0.784
EM	0.793, 0.821, 0.870, 0.817	0.895	0.682	0.826
PG	0.694, 0.774, 0.825, 0.776	0.852	0.591	0.769
PR	0.760, 0.695, 0.702, 0.665	0.799	0.499	0.706
PL	0.873, 0.892, 0.861, 0.806	0.918	0.737	0.859
SA	0.719, 0.899, 0.833, 0.823	0.890	0.670	0.818

Note. Dimension abbreviations same as those in Table 1. CR ≥ 0.70 [58], AVE ≥ 0.50 [45]

As shown in Table 4, the hierarchical model conceptualized psychological well-being (PWB) as a second-order latent construct encompassing six first-order dimensions: AU, EM, PG, PL, PR, and SA. All standardized loadings from the second-order factor to its associated subcomponents were significant at *p* = 0.001, with values ranging from 0.712 (PWB → AU) to 0.896 (PWB → PG). The 95% bias-corrected confidence intervals excluded zero, indicating that these estimates were both stable and reliable. The R² values for the first-order factors ranged between 0.506 and 0.803, suggesting that the higher-order construct explained a substantial amount of variance in each subdimension.

**Table 4:** The parameter estimates of the hierarchical model

Parameter	Standardized estimate	Bias-corrected 95% CI	*p*	*R*^2^
PWB→ AU	0.712	[0.438, 0.579]	0.001	0.506
PWB→ EM	0.806	[0.578, 0.715]	0.001	0.650
PWB→ PG	0.896	[0.739, 0.862]	0.001	0.803
PWB→ PL	0.780	[0.538, 0.675]	0.001	0.608
PWB→ PR	0.760	[0.499, 0.658]	0.001	0.578
PWB→ SA	0.890	[0.737, 0.847]	0.001	0.793

Dimension abbreviations same as those in Table 1

While the hierarchical model offered a theoretically coherent structure and yielded acceptable parameter estimates, its overall fit indices were marginally lower than those of the six-factor correlated model, reflecting a typical trade-off between parsimony and model fit.

Convergent validity was assessed using CR and AVE. CR values ranged from 0.799 to 0.918 across the six dimensions, exceeding the commonly recommended threshold of 0.70. AVE values ranged from 0.499 to 0.737. Although the AVE for Positive Relations fell just below the 0.50 cutoff, its CR of 0.799 suggests that the construct still possesses satisfactory convergent validity. As noted by Fornell and Larcker (1981), a construct may be deemed valid even with a slightly lower AVE, provided its CR exceeds 0.60 [45].

We assessed discriminant validity by comparing each construct’s AVE square root with its correlations with other factors. Following the criterion proposed by Fornell and Larcker [45], we found that the square roots of the AVEs (ranging from 0.706 to 0.859) consistently surpassed the inter-construct correlations. For example, the AU’s AVE square root was higher than its strongest correlation with another factor (*r* = 0.593 with EM), a pattern observed across all dimensions. These results support the distinctiveness of the latent constructs. The detailed outcomes are presented in Table 5.

**Table 5:** Pearson correlation matrix and discriminant validity based on the Fornell–Larcker criterion

	PWBS (Total)	AU	EM	PG	PR	PL	SA
AU	0.752^**^	**0.784**					
EM	0.805^**^	0.593^**^	**0.826**				
PG	0.846^**^	0.548^**^	0.695^**^	**0.769**			
PR	0.736^**^	0.375^**^	0.452^**^	0.575^**^	**0.706**		
PL	0.812^**^	0.494^**^	0.535^**^	0.610^**^	0.571^**^	**0.859**	
SA	0.866^**^	0.578^**^	0.625^**^	0.689^**^	0.608^**^	0.659^**^	**0.818**
	Correlation with external scales						
ASES (Total)	0.590^**^	0.454^**^	0.451^**^	0.479^**^	0.431^**^	0.521^**^	0.498^**^

Dnimension abbreviations same as those in Table 1. ^**^p < 0.01; *N* = 855. Bold values on the diagonal indicate the square roots of the average variance extracted (√AVE) for each construct

Overall, the CFA results provide compelling evidence for the construct validity of the Brief Chinese PWBS, demonstrating sound convergent and discriminant validity.

### Criterion-related validity

We assessed criterion-related validity by computing Pearson correlation coefficients between the ASES mean score and the total as well as subscale scores of the PWBS. As shown in Table 5, the total PWBS score was strongly and positively correlated with ASE (*r* = 0.590, *p* < 0.001), providing robust evidence of criterion validity. All six PWBS subscales were also significantly associated with ASES scores: AU (*r* = 0.454, *p* < 0.001), EM (*r* = 0.451, *p* < 0.001), PG (*r* = 0.479, *p* < 0.001), PR (*r* = 0.431, *p* < 0.001), PL (*r* = 0.521, *p* < 0.001), and SA (*r* = 0.498, *p* < 0.001). These findings indicate that higher levels of PWB are consistently linked to stronger ASE among Chinese adolescents.

To further examine the criterion-related validity of the Brief Chinese PWBS, we performed a linear regression analysis using ASE as the dependent variable. The total PWBS score significantly predicted ASE (β = 0.59, *p* < 0.001), accounting for 34.8% of the variance (R² = 0.348). These results indicate that PWB, as assessed by the PWBS, meaningfully contributes to adolescents perceived academic competence, both at the overall level and within its individual dimensions.

### Measurement invariance across gender and age groups

We conducted a multi-group CFA to assess measurement invariance of the proposed six-factor model across gender (male vs. female) and age groups (15–16 vs. 17–18 years). The analysis examined three levels of invariance: configural, metric, and scalar. Nested model comparisons showed support for all levels of invariance, with changes in fit indices falling within the recommended thresholds (ΔCFI ≤ 0.010, ΔRMSEA ≤ 0.015; [59]). Detailed findings are reported in Table 6.

**Table 6:** Model fit indexes for multi-group analysis of gender differences

Group	Model	χ^2^ (df)	CFI	TLI	SRMR	RMSEA	Comparison	Δχ2 (Δdf)	*p*-value	ΔCFI
Gender	M1: Baseline-Male	574.017 (234)^***^	0.948	0.939	0.048	0.061	—	—	—	—
	M2: Baseline-Female	673.272 (234)^***^	0.942	0.932	0.053	0.063				
	M3: Configural invariance	1247.294 (468)^***^	0.945	0.935	0.048	0.044	—	—	—	—
	M4: Metric invariance	1268.561(486)^***^	0.945	0.937	0.047	0.043	M4-M3	21.267(18)	0.269	0.000
	M5: Scalar invariance	1296.873(507)^***^	0.944	0.939	0.054	0.043	M5-M4	28.312(21)	0.129	−0.001
	M6: Fully constrained model	1327.605(534)^***^	0.944	0.942	0.056	0.042	M6-M5	30.732(27)	0.283	0.000
Age	M1: Baseline-Age15-16	569.699 (234)^***^	0.957	0.950	0.042	0.055	—	—	—	—
	M2: Baseline- Age17-18	595.112 (234)^***^	0.943	0.933	0.053	0.064				
	M3: Configural invariance	1516.450 (474)^***^	0.926	0.914	0.046	0.051	—	—	—	—
	M4: Metric invariance	1533.922(492)^***^	0.926	0.917	0.047	0.047	M4-M3	17.474(18)	0.491	0.000
	M5: Scalar invariance	1564.216(513)^***^	0.926	0.920	0.052	0.046	M5-M4	30.294(21)	0.086	0.000
	M6: Fully constrained model	1607.196(537)^***^	0.924	0.922	0.052	0.046	M6-M5	42.980(24)^*^	0.010	−0.002

As shown in Table 6, the six-factor model exhibited acceptable configural invariance across male and female participants (M3: χ²(474) = 1516.45, CFI = 0.926, TLI = 0.914, RMSEA = 0.051, SRMR = 0.046), indicating that both groups share the same factor structure. When factor loadings were constrained equal (metric model, M4: χ²(492) = 1533.92, CFI = 0.926, ΔCFI = 0.000), fit remained stable, supporting metric invariance. Further constraining item intercepts (scalar model, M5: χ²(513) = 1564.22, CFI = 0.926, ΔCFI = 0.000) also produced negligible change in fit, indicating scalar invariance and justifying comparison of latent means across gender. Finally, constraining residual variances (strict model, M6: χ²(537) = 1607.20, CFI = 0.924, ΔCFI= − 0.002) yielded a small drop in CFI; however, given that ΔCFI remains within the 0.01 threshold, strict invariance may be deemed tenable, suggesting similar measurement precision across genders.

Table 6 also reports multi-group CFA across two age cohorts (15–16 vs. 17–18 years). The configural model (M3: χ² (474) = 1516.45, CFI = 0.926, TLI = 0.914, RMSEA = 0.051, SRMR = 0.046) confirmed that both age groups shared the same six-factor structure. Imposing equality constraints on factor loadings (metric invariance, M4: χ² (492) = 1533.92, CFI = 0.926, ΔCFI = 0.000) did not diminish fit, supporting equivalent item loadings across ages. Equating item intercepts (scalar invariance, M5: χ² (513) = 1564.22, CFI = 0.926, ΔCFI = 0.000) likewise maintained model fit, allowing for valid latent mean comparisons. However, full residual invariance (M6: χ² (537) = 1607.20, CFI = 0.924, ΔCFI=–0.002) resulted in a statistically significant χ² increase (*p* = 0.010), indicating that strict invariance does not hold; residual variances differ between the two age groups. Nonetheless, attainment of scalar invariance suffices for meaningful group comparisons of the PWB latent constructs.

## Discussions and implications

Assessing psychological well-being is pivotal for understanding both mental health problems and positive functioning. However, despite the widespread use of Ryff’s model across cultures, evidence on the cross-cultural and developmental equivalence of its brief Chinese adaptation remains scarce. Most previous validations of Ryff’s PWBS or its shortened versions have focused on Western or adult populations, leaving a gap in understanding how this eudaimonic construct operates among Chinese adolescents. Addressing this limitation, the present study provides one of the first systematic examinations of the factorial validity and measurement invariance of the Brief Chinese PWBS (BC-PWBS) in a large adolescent sample from mainland China. In the Chinese context, the construct of psychological well-being (PWB) is especially relevant given the increasing emphasis on holistic mental health promotion in education and society.

The results of this study extend previous research conducted primarily in adult or Hong Kong samples and highlight the scale’s robustness for use in diverse adolescent populations. Overall, the findings provide robust evidence for its psychometric soundness, supporting its use as a reliable and theoretically grounded measure of psychological well-being in this population. Both Cronbach’s α and McDonald’s ω indicated excellent internal consistency across all six subscales and the total scale, consistent with prior adolescent validations [19, 20] and exceeding some adult validations [15]. This strong reliability further underscores the psychometric robustness of the brief PWBS for adolescent populations. According to the *Standard for Educational and Psychological Testing* [57], CFA provides evidence based on internal structure, supporting the theoretical organization of the scale dimensions. Specifically, the CFA supported the theoretical six-factor structure proposed by Ryff [6], with all standardized factor loadings surpassing the commonly accepted cutoff of 0.60 [58]. Although the fit indices of the second-order model were slightly lower than those of the correlated six-factor model, it remained conceptually meaningful in representing psychological well-being as a higher-order construct comprising six interrelated dimensions [8]. Although the correlations among the six dimensions were relatively strong, this pattern is theoretically justifiable, as these dimensions represent interrelated yet distinct aspects of psychological well-being (PWB). Consistent with Furr [60], the strong correlations indicate that the dimensions share a common psychological meaning while maintaining conceptual distinctions. To further examine this possibility, a bi-factor model was tested and demonstrated superior fit (CFI = 0.955, RMSEA = 0.059), suggesting the presence of a general PWB factor alongside the six specific dimensions. The slightly elevated SRMR may reflect the complexity of the bifactor model, rather than a substantive lack of fit. Therefore, both the multidimensional and general-factor representations of PWB are meaningful and complementary. Taken together, this dual support strengthens the argument for the multidimensional yet unified nature of psychological well-being in Chinese adolescents. This result reinforces the theoretical coherence of Ryff’s multidimensional model and suggests that the core components of well-being are interpretable within Chinese collectivistic cultural contexts. Furthermore, convergent and discriminant validity were also supported. CR values for all subscales exceeded the 0.70 criterion, and most AVE values surpassed 0.50. Although the AVE for Positive Relations was marginally below 0.50, the corresponding CR of 0.842 compensates for this limitation, which aligns with the argument that high CR can justify a lower AVE [45]. The AVEs’ square roots were higher than inter-factor correlations across all constructs, supporting discriminant validity.

Additionally, the positive correlations between PWBS and ASES further confirm the criterion-related validity of the scale. All six dimensions were significantly associated with ASES, consistent with prior findings that PWB is positively related to motivation and self-beliefs in academic settings [1, 19]. These results suggest that adolescents who report greater well-being also tend to perceive themselves as more efficacious learners. According to Bandura’s (1997) self-efficacy theory, students who perceive themselves as capable and effective in academic settings tend to experience stronger motivation, competence, and mastery—all essential features of psychological well-being [29]. From the perspective of self-determination theory [30, 31], academic self-efficacy also fulfills adolescents’ basic psychological needs for competence, autonomy, and relatedness. This alignment provides strong theoretical support for using academic self-efficacy as an external criterion and highlights the role of academic confidence as a salient source of well-being during adolescence.

Finally, the current study tested measurement invariance across gender and age categories and found evidence for configural, metric, and scalar invariance. These findings suggest that the scale operates similarly across male and female adolescents, as well as across younger and older age groups (15–16 vs. 17–18 years), supporting its utility for group comparisons. Although full residual invariance was not achieved in age-based analysis, the attainment of scalar invariance is sufficient to justify comparison of latent means [48]. By examining measurement invariance across gender and age groups, this study contributes to the cross-cultural applicability of the PWBS framework and provides a foundation for future large-scale or comparative research on adolescent well-being in China.

The present findings can be compared with validations of Ryff’s PWBS in other Chinese and Asian contexts. For example, Chan et al. (2019) confirmed the six-factor model among Hong Kong adolescents [19], and related studies in Japan [61] and Singapore [62] also supported the multidimensional structure, indicating a degree of cultural universality. Nevertheless, cultural nuances are evident. Although Hong Kong and mainland China share linguistic and Confucian roots, Hong Kong adolescents are exposed to more Westernized and individualistic educational influences, which may lead to different readings of autonomy and life purpose. From a psychological perspective, autonomy in collectivistic societies is often enacted through relational interdependence rather than personal independence [63], so that autonomy may be understood as the capacity to maintain social harmony and fulfill role expectations. This interpretation echoes cross-cultural findings that autonomy and purpose can be construed relationally in collectivistic contexts [64]. In contrast, mainland Chinese adolescents may derive well-being more strongly from academic competence and relational harmony, reflecting Confucian emphases on self-cultivation and social obligation [65, 66]. These culturally informed mechanisms may explain the relatively stronger loadings for Positive Relations and Environmental Mastery observed here. Overall, the consistent factorial validity across contexts suggests Ryff’s six dimensions are broadly applicable yet locally contextualized. This cultural differentiation also aligns with Hofstede’s collectivism–individualism dimension, whereby relational harmony can serve as a central route to adolescent well-being [67].

Building on the scale’s robust psychometric performance, this study establishes a baseline for future cross-cultural comparisons and offers a concise, reliable tool for research and practice. Its practical and theoretical implications warrant further consideration. The validated BC-PWBS offers researchers and educators a concise, psychometrically sound instrument for assessing psychological well-being in school settings. Its demonstrated stability across gender and age groups enhances its suitability for comparative and longitudinal use. In educational practice, the scale may inform school-based well-being programs, intervention evaluation, and mental health monitoring, contributing to the promotion of positive youth development within Chinese cultural contexts. The scale can be used to monitor students’ well-being, identify those at risk, and evaluate the effectiveness of mental health or well-being interventions. Given the increasing emphasis on promoting positive development and mental health among adolescents in China [32, 33, 68, 69], the availability of a reliable and valid tool such as the brief PWBS is timely and valuable for both researchers and practitioners. Theoretically, this study supports the cross-cultural applicability of Ryff’s multidimensional model of eudaimonic well-being. The six core dimensions, including autonomy, environmental mastery, personal growth, positive relations, purpose in life, and self-acceptance, appear to be relevant and meaningful to Chinese adolescents, suggesting that PWB can be conceptualized as a functional and multidimensional construct across cultural contexts. The significant correlation between PWB and ASE further highlights the link between emotional and motivational processes in adolescent development.

### Limitations and future directions

Several limitations warrant consideration. First, our adolescent sample was drawn exclusively from senior high schools in a single city in northeastern China, potentially limiting the generalizability of the findings. Subsequent research could extend this work by recruiting a more geographically and developmentally diverse cohort, for example, by including junior middle school students and adolescents from multiple regions, to ensure broader applicability of the scale. Second, the cross-sectional nature of the research means that data were collected at only one point in time, preventing an assessment of predictive validity or test-retest reliability. This limits the ability to evaluate the stability of the scale over time. Future research could adopt longitudinal designs with extended time spans and multiple measurement points to better understand the temporal stability of the PWBS. Another limitation is that the present study relied on a single-method self-report design. Since validation is an ongoing process, future research may consider adopting a multi-trait multi-method (MTMM) approach—such as incorporating observer ratings or behavioural coding—provide a more comprehensive evaluation of the scale’s validity. In addition, the use of a single criterion variable (academic self-efficacy) to establish criterion-related validity may also limit the comprehensiveness of the validation evidence. Future studies could incorporate multiple external correlates, such as life satisfaction, academic engagement, or mental health indicators, to provide a more comprehensive assessment of criterion validity.

In addition, although the present validation focused on adolescents, the theoretical foundation and psychometric robustness of the PWBS suggest that it may have potential applicability to other populations, such as college students or emerging adults. However, as psychological well-being may manifest differently across developmental stages, the use of the scale in other age groups should be approached with caution. Future research is therefore encouraged to further examine the validity, reliability, and measurement invariance of the PWBS across diverse populations before broader application.

Despite certain limitations, this study offers several important strengths. As the first study to comprehensively assess the brief Chinese PWBS in a mainland Chinese adolescent population, this work fills a notable gap in the literature. Given that adolescents undergo distinct developmental changes, it is important to validate such measures in this specific age group. Recognizing that psychological well‑being may manifest differently across developmental stages, we explicitly tested and established measurement invariance across both gender and age groups (Grades 10 vs. 11), confirming that the scale functions equivalently for boys and girls and for younger versus older high‑school students. This contribution ensures confidence in comparing scores across these key subgroups. Moreover, the brevity and clarity of the scale make it suitable for use in large-scale surveys and school-based settings.

## Conclusion

This study offers compelling evidence for the psychometric validity of the BC-PWBS among adolescents in mainland China. Evidence from confirmatory factor analysis and multi-group tests supported the factorial validity and measurement invariance of the scale across gender and age groups, indicating its psychometric stability across key demographic subgroups. The scale also showed strong criterion-related validity through its significant positive correlations with ASE. Overall, the BC-PWBS demonstrated satisfactory reliability, clear factorial validity, meaningful criterion correlations, and configural and metric invariance across gender and age, suggesting that Ryff’s conceptualization of psychological well-being remains meaningful in collectivistic educational settings. These findings suggest that the BC-PWBS is a psychometrically robust tool for assessing psychological well-being in Chinese adolescents. Its brevity and theoretical coherence make it suitable for both large-scale surveys and cross-cultural research. Future studies could further examine its longitudinal invariance and explore its predictive validity with other indicators of youth adjustment and mental health across different age and cultural groups, thereby further evaluating the universality of Ryff’s eudaimonic framework.

## Supplementary Information

Supplementary Material 1

## Data Availability

The datasets used in this study are available from the first author upon reasonable request.
