# Supplementary material for: Psychometric validation of the brief Chinese psychological well-being scale in a sample of Mainland Chinese adolescents
Source: BMC Psychol. 2026 Jan 9;14:290. doi: 10.1186/s40359-025-03952-x (PMC12954903; doi:10.1186/s40359-025-03952-x)
Supplement: Supplementary file 1 — Supplementary Material 1 [file 40359_2025_3952_MOESM1_ESM.docx]

**Brief Chinese Psychological Well-Being Scale (BC-PWBS)**

|  | **items** | **Strongly disagree** | **Disagree** | **Neutral** | **Agree** | **Strongly agree** |
| --- | --- | --- | --- | --- | --- | --- |
| 1 | 即使我的看法与大多数人相反，我也不怕说出来。I am not afraid to voice my opinions, even when they are in opposition to the opinions of most  people. | 1 | 2 | 3 | 4 | 5 |
| 2 | 我的决定通常不受别人影响。My decisions are not usually influenced by what everyone else is doing. | 1 | 2 | 3 | 4 | 5 |
| 3 | 我对自己的看法有信心，即使这些看法与大众相反。I have confidence in my opinions, even if they are contrary to the general consensus. | 1 | 2 | 3 | 4 | 5 |
| 4 | 我以自己认为重要的标准来评价自己，而不是根据别人的价值观。I judge myself by what I think is important, not by the values of what others think is important. | 1 | 2 | 3 | 4 | 5 |
| 5 | 一般来说，我觉得我能管好自己的生活状况。In general, I feel I can manage the situation in which I live. | 1 | 2 | 3 | 4 | 5 |
| 6 | 我能处理好自己日常生活很多职责。I am quite good at managing the many responsibilities of my daily life. | 1 | 2 | 3 | 4 | 5 |
| 7 | 为自己安排一种满意的生活，我并不感到困难。I don’t have difficulty arranging my life in a way that is satisfying to me. | 1 | 2 | 3 | 4 | 5 |
| 8 | 我能为自己安排一种我喜欢的生活模式和环境。I am able to arrange a living environment and a lifestyle for myself that is much to my liking. | 1 | 2 | 3 | 4 | 5 |
| 9 | 有新经历去激发我们思考自己和世界，我认为是重要的。I think it is important to have new experiences that challenge how you think about yourself and the world. | 1 | 2 | 3 | 4 | 5 |
| 10 | 回想过去这些年，我进步良多。When I think about it, I have improved much as a person over the years. | 1 | 2 | 3 | 4 | 5 |
| 11 | 我感到自己这些年来有很多成长。I have the sense that I have developed a lot as a person over time. | 1 | 2 | 3 | 4 | 5 |
| 12 | 对我来说，生命是一个不断学习、改变和成长的过程。For me, life has been a continuous process of learning, changing, and growth. | 1 | 2 | 3 | 4 | 5 |
| 13 | 很多人觉得我有爱心和充满情感。Most people see me as loving and affectionate. | 1 | 2 | 3 | 4 | 5 |
| 14 | 我享受与家人和朋友交谈和互诉心事。I enjoy personal and mutual conversations with family members or friends. | 1 | 2 | 3 | 4 | 5 |
| 15 | 其他人形容我是一个肯付出、愿意为别人花时间的人。People would describe me as a giving person, willing to share my time with others. | 1 | 2 | 3 | 4 | 5 |
| 16 | 我知道我可以信任朋友，他们也知道可以信任我。I know that I can trust my friends, and they know they can trust me. | 1 | 2 | 3 | 4 | 5 |
| 17 | 我有生活方向和目标。I have a sense of direction and purpose in life. | 1 | 2 | 3 | 4 | 5 |
| 18 | 我清楚自己的人生要成就什么。I have a good sense of what it is I'm trying to accomplish in life. | 1 | 2 | 3 | 4 | 5 |
| 19 | 我喜欢为未来制定计划并努力去实现它。I enjoy making plans for the future and working to make them a reality. | 1 | 2 | 3 | 4 | 5 |
| 20 | 有些人漫无目的地生活，但我并不是这样。Some people wander aimlessly through life, but I am not one of them. | 1 | 2 | 3 | 4 | 5 |
| 21 | 回顾自己的生活故事，我对那些经历和结果感到高兴。When I look at the story of my life, I am pleased with how things have turned out. | 1 | 2 | 3 | 4 | 5 |
| 22 | 整体来说，我对自己有信心和肯定自己。In general, I feel confident and positive about myself. | 1 | 2 | 3 | 4 | 5 |
| 23 | 我喜欢自己大部分的性格。I like most parts of my personality. | 1 | 2 | 3 | 4 | 5 |
| 24 | 与朋友和其他相识的人相比，我对自己感到满意。When I compare myself to friends and acquaintances, it makes me feel good about who I am. | 1 | 2 | 3 | 4 | 5 |
